# Position-dependent urinary retention in a traumatic brain injury patient: a case report

**DOI:** 10.1186/1757-1626-2-9120

**Published:** 2009-11-30

**Authors:** Ivan Chernev, Kun Yan

**Affiliations:** 1 Department of Rehabilitation Medicine, Boston University Medical Center, Boston, Massachusetts, USA; 2 Department of Physical Medicine and Rehabilitation, Veterans Health Administration, Boston Healthcare System, West Roxbury, Massachusetts, USA

## Abstract

**Introduction:**

Voiding disorders are common complication after traumatic brain injury. Usually, they are caused by neurogenic bladder although they can also occur as a result of other pathologic processes and conditions as well as side effects of medications.

**Case presentation:**

A 62-year-old traumatic brain injury patient with position-dependent urinary retention is presented in this article. Neurogenic bladder with detrusor sphincter dyssynergia was suspected initially, with detection of multiple small bladder stones as the final cause of his urinary retention afterwards.

**Conclusion:**

Careful clinical, imaging, and urodynamic evaluation must be performed in traumatic brain injury patients to exclude the coexistence of two or more factors leading to urinary dysfunction in this population group.

## Introduction

Voiding disorders are very common after traumatic brain injury (TBI) [1,2]. Incontinence and urinary retention can cause urinary tract infections (UTI's), development of skin ulcers, formation of stones and renal failure. They are also predictive factors for prolonged rehabilitation period with less favorable outcome and can lead to social embarrassment and isolation. Most of the voiding disorders in TBI patients are caused by neurogenic bladder although they can also occur as a result of other common or less common etiologies such as overuse of narcotics, use of medications with anthicholinergic effect, clogged catheters or benign hypertrophic prostate [1-3].

The aim of this article is to present an unusual case of position-dependent urinary retention in a TBI patient due to multiple small bladder stones and to underline the fact that different etiologies of voiding disorders may sometimes coexist.

## Case description

A 62-year-old white man with a previous medical history of hypertension, hypercholesterolemia, dyslexia and severe TBI with prolonged, multiple-admission, acute and rehabilitation hospitalization course was admitted to the authors' acute rehabilitation unit 4 months after he fell off a 16-foot ladder while working on the roof of his house. The initial Glasgow Coma Scale was 13. The patient suffered a large right-sided extra-occular hemorrhage with subarachnoid extension and 11 millimeters leftward shift, subfalcine herniation and compression of the right lateral ventricle. There was also a right uncal herniation, multiple foci of hemorrhage, and contusion of the left parietal cortex. In addition, he had a longitudinal fracture of the left temporal and left parietal bones. He underwent right-sided craniotomy and evacuation with right-sided temporal lobectomy and duraplasty. Due to developing hydrocephalus of the left lateral and third ventricles a ventriculoperitoneal shunt was placed prior to his transfer to the author's facility. Since his accident the patient has been diagnosed with recurrent multi-drug resistant UTI's, which required antibiotic treatment.

Prior to admission to the author's rehabilitation unit, the patient's urinary output was managed by indwelling Foley catheter, which was discontinued shortly after his admission. A condom catheter was applied for management of the bladder and, the patient was noticed to have periods with complete empting of the bladder interchanged with periods of retention with increased postvoid residual volums and the need for intermittent catheterization. Urodynamic study was requested and showed severe detrusor overactivity but did not show definite detrusor sphincter dyssynergia (DSD).

Physical exam revealed a patient with quadriparesis with the left side more affected than the right one in no acute distress, lying in bed not following commands. Vital signs were within normal physiological limits. Abdomen was soft, non-tender and non-distended. Penis was with circumcized phallus, and meatus was patent. Scrotum was with no evidence of erythema or edema. Testicles were descended bilaterally.

Labs: white blood cell count: 6400 cells/cubic milliliter, hematocrit: 27.1%, prostate specific antigen: 9 nanograms/milliliter, creatinine: 0.6 milligram/deciliter, urea nitrogen 24 milligram/deciliter. Urinalysis: cloudy urine with trace of leukocyte esterase, 14 white blood cell/high-power field, 1 red blood cell/high-power field and a few amorphous crystals.

Medications: Acetaminophen, Aspirin, Bisacodyl, Docusate, Enoxaparin, Ferrous sulfate, Lisinopril, Metoprolol, Milk of magnesia, Multivitamins, Omeprazole, Phenytoin, Senna, Terazosin, Thiamin, and Valproic acid.

Consequently, it was also noted that the patient had good urinary output while in supine position but very limited or no urinary output at all while positioned in the wheelchair.

**Figure 1:**
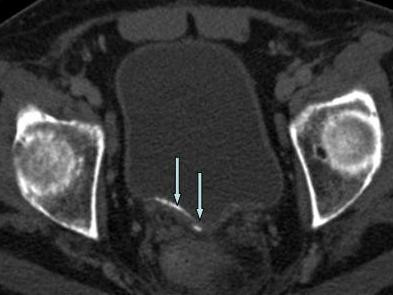
**Axial pelvic CT scan with contrast**. Note the linear calcific densities within the dependent portion of the urinary bladder (arrows).

**Figure 2:**
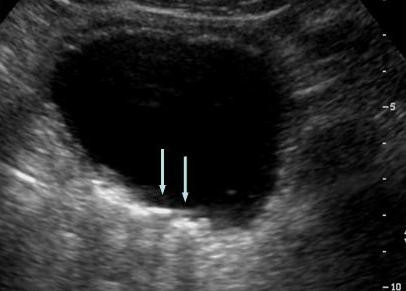
**Abdominal ultrasound**. Note the linear calcifications in the posterior wall of urinary bladder with posterior acoustic shadowing (arrows).

**Figure 3:**
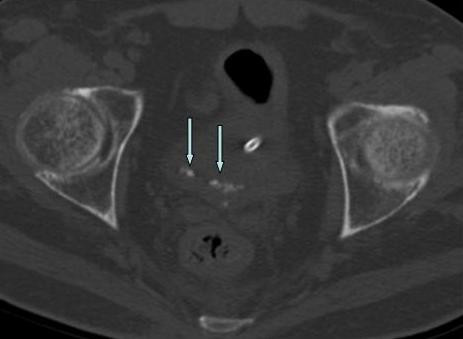
**Axial pelvic CT scan without contrast**. Note the multiple calcifications located in the dependent portion of the bladder demonstrating interval change in appearance (arrows).

**Figure 4:**
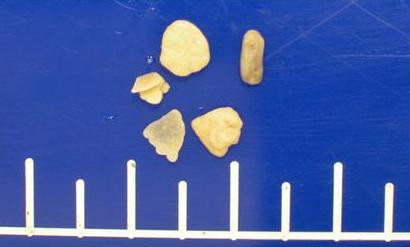
**Macroscopic pathology slide**. Small yellow stones measuring from 3 to 5 millimeters in diameter.

To evaluate this position-dependent urinary retention an abdominal computed tomography (CT) scan was performed and showed multiple nondependent calcific densities within the dependent portion of the urinary bladder, which were read by radiologist as multiple bladder stones versus bladder wall calcifications (Figure 1). No renal or ureteral calculi were noticed. Ultrasound, completed the next day, reveled linear calcifications in the posterior wall of urinary bladder with posterior acoustic shadowing. Due to patient's inability to cooperate, it was difficult to define if these calcifications were within the wall or were due to stones (Figure 2). Urology was consulted again and they suggested a neurogenic bladder etiology with calcified debris in the bladder and recommended continuing Foley catheter for bladder management. Subsequently, a follow up CT scan was done and showed dependent bladder calcifications demonstrating interval change in appearance on decubitus imaging and change in appearance when compared to the patient's prior CT scan (Figure 3). A cystoscopy was performed by urology the same day after the CT scan with the finding of approximately 15 small stones measuring 3 to 5 millimeters in diameter. There were no murky debris anticipated and no other worrisome lesions. All of the stones were irrigated out through the sheath of the resectoscope (Figure 4).

In the following days after the procedure the patient was able to spontaneously urinate in both sitting and recumbent position without high postvoiding residuals (less than 60 milliliters) and need for intermittent catheterization. Condom catheter was used in the post cystoscopy period and bladder training was initiated as well.

## Discussion

A spectrum of voiding disorders with different pathology and pathophisiology in TBI patients may lead to urinary incontinence or retention. Incontinence is much more frequently encountered than retention [1,2]. Typically, TBI patients present with a hyperactive neurogenic bladder, with uninhibited detrusor reflex in which the bladder volume is reduced but empties completely with normal postvoiding intravesicular volumes [1-3].

Less frequently TBI patients may present with urinary retention. This may be due to detrusor sphincter dyssynergia (DSD) in which there is dysbalance of detrusor and sphincter muscles. Other more easily reversible causes such as clogged catheters or medication side effects can also possibly cause urinary retention and have to be ruled out first before diagnostic and treatment approach for neurogenic bladder is initiated. In older patients the possibility of benign hyperplasia of the prostate has to be considered as well. Finally, urinary retention may be the result of urinary tract stones. Although, bladder calculi are now less frequently encountered in United States they can be found occasionally in TBI patients. In the general population, bladder stones are usually associated with bladder outlet obstruction, urinary infections, radiation, schistosomiasis, bladder diverticuli and foreign bodies such as Foley catheters and prostatic urethral stents [4,5]. The vesical stones may be single or multiple with variation in incidence reported from different authors [5,6]. In TBI patients, prolonged catheterization, retention of urine as a result of neurogenic bladder, and UTI may predispose to formation of bladder stones. Even though bladder stones may be completely asymptomatic, more often they present with suprapubic pain, dysuria, intermittency, hematuria, frequency, hesitancy and nocturia. They may also present with sudden termination of the ability to void due to a lodged stone in the urethra [6,7].

In this clinical case the patient did not have a previous history of bladder stones and benign prostate hyperplasia and initially it was thought that the bladder dysfunction is related to neurogenic bladder. As his retention was intermittent with periods of full empting interchanged with retention and in the beginning not noticed to be related to his position, neurogenic bladder was high on the differential list, which was not confirmed by the urodynamic studies. Subsequently, it was found that the patient had very limited or no urinary output in sitting and upright position and increased urinary output in lying position. In contrast to this patient a study demonstrated that urinary flow is decreased in the recumbent relative to the standing position [8]. That finding may suggest that vertical body position may facilitate micturition likely due to gravitational forces. In fact, the urinary retention in this patient was caused by multiple small stones and was related to the position of the patient. In recumbent position the urinary stones were moving away from the urinary bladder neck allowing the urine to drain freely. In sitting or standing position, following the gravity, the stones were repositioning to the bladder neck, aggregating on the top of the internal urethral orifice mechanically blocking the passage of urine. This patient did not have a stone lodged into the urethra causing acute complete obstruction of the urinary flow but rather had intermittent gravity- and position-dependent urinary obstruction as a result of multiple small bladder stones intermittently blocking the urine flow. Initially, it was difficult to differentiate these stones from bladder wall calcifications because of the patient immobility as well as the small size of the stones positioned on the bladder wall surface and imitating wall calcifications. After cystoscopy with removal of 15 stones from the bladder this patient was able to pass urine in the condom catheter in any position. His UTI was cleared and his mental status improved significantly.

Occasionally, other causes of voiding disorders may be superimposed on neurogenic bladder. In this case bladder stones causing urinary retention were coexistent with previously diagnosed hyperactive bladder without DSD. It is important that clinicians look for and exclude other etiologies before considering the neurogenic bladder as the single explanation for urinary voiding dysfunction in a TBI patient even though previous diagnosis has been made.

Timely diagnosis of other less common causes for voiding disorders, sometimes superimposed on underlying neurogenic bladder, may prevent additional morbidity and mortality and avoid unnecessary tests.

## Abbreviations

TBI: traumatic brain injury; UTI: urinary tract infection: DSD: detrusor sphincter dyssynergia; CT: computed tomography

## Consent

Written informed consent was obtained from the patient for publication of this case report and accompanying images. A copy of the written consent is available for review from the journal's Editor-in-Chief.

## Competing interests

The authors declare that they have no competing interests.

## Authors' contributions

IC and KY participated in the collection of data, drafting and critically reviewing the manuscript.
